# Imaging manifestations in infantile GM1 gangliosidosis: a rare lysosomal storage disorder: a paediatric case report

**DOI:** 10.1093/bjrcr/uaaf009

**Published:** 2025-04-01

**Authors:** Shreya Bhat, Sachin Sharma, Sunil Bhat, Anjana Kaul

**Affiliations:** Department of Radiodiagnosis, NMCH Rohtas, Sasaram, Bihar 821305, India; Department of Radiodiagnosis, NMCH Rohtas, Sasaram, Bihar 821305, India; SL Diagnostic Center, Jammu 180012, India; Divine Healthcare, Jammu 180012, India

**Keywords:** GM1 gangliosidosis, lysosomal storage disease, dermal melanocytosis, GTCS (generalized tonic-clonic seizure), hypomyelination, thalamus, NCCT

## Abstract

Mono-sialo-tetra-hexosylganglioside, also known as infantile GM1 gangliosidosis, is an autosomal recessive lysosomal storage disorder caused by a mutation in the GLB1 gene that stops the β-galactosidase enzyme from working. We have discussed a case of infantile GM1 gangliosidosis which presented with abnormal body movements, extensive dermal melanocytosis over back and gluteal region, coarse facial features, and macrocephaly. Radiological features included antero-inferior beaking of second, third, and fourth lumbar vertebrae, bilateral hyperdense thalami on non-contrast CT. On T2-weighted images, there is a persistently high signal intensity of the white matter and subcortical U fibres, which indicates bilateral bulky thalami with T2 hypointense and significantly impaired myelination. Reduced β-galactosidase activity verified the diagnosis.

## Introduction

A mutation in the GLB1 gene on chromosome 3p22.3 causes infantile GM1 gangliosidosis, also known as mono-sialo-tetra-hexosylganglioside, a rare autosomal recessive lysosomal storage disorder. The β-galactosidase enzyme, which stops gangliosides from being broken down, is reduced or eliminated by this mutation. As a result, gangliosides accumulate in various organ systems, especially the neurological system, where they eventually destroy nerve cells.^1^

Infantile GM1 gangliosidosis is characterized by increasing spastic, cerebellar, and extrapyramidal symptoms along with mental decline; facial dysmorphisms. Only a small number of cases have been reported neuroimaging results.^2^

The disease’s most severe manifestations will show signs of oligosaccharidosis, mucopolysaccharidosis, and neuronal lipidosis. Deficient β-galactosidase activity in enzyme assays is the only surefire method of diagnosing GM1 gangliosidosis. GM1 gangliosidosis is thought to affect 1 in 100 000 to 200 000 live births.^3^

## Case report

A 12-month-old girl child who was born to non-consanguineous parents presented with complaints of bluish discolouration on her hands and lips as well as unusual body motions. She experienced GTCS (generalized tonic-clonic seizure) involving both upper and lower limbs, uprolling of eyes, and drooling of saliva. Additionally, she showed signs of global developmental delay, including chronic bidextrous reach, failure to sit with or without support, and lack of neck holding, all of which were linked to generalized hypotonia.

**Figure 1.**
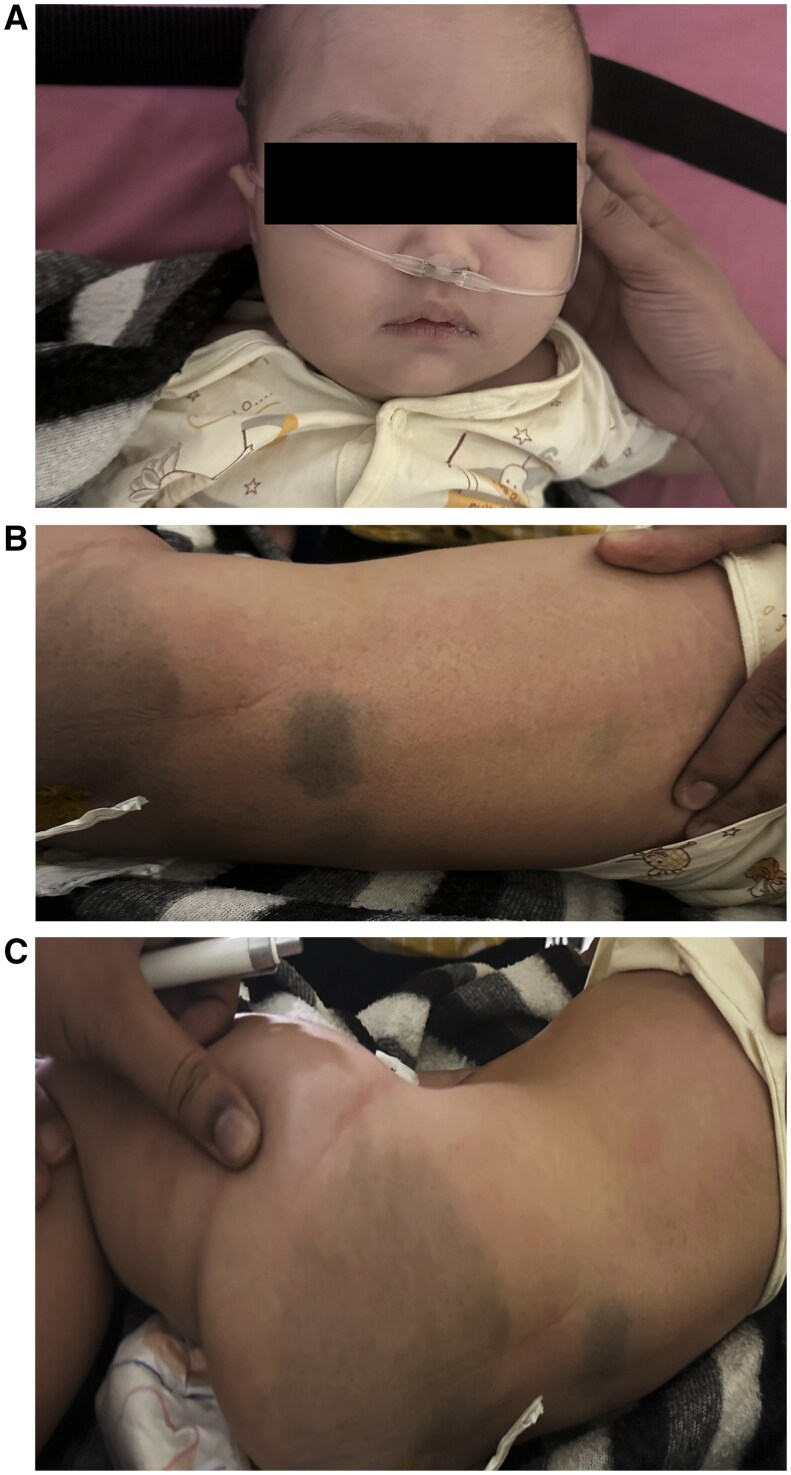
(A) Coarse facial features in a 12-month-old infant. (B and C) Extensive dermal melanocytosis of back and gluteal region.

Upon physical examination, the back and gluteal region showed variable sized bluish pigmentation which suggested significant cutaneous melanocytosis (Figure 1B and C), lowset ears, coarse facial features, a depressed nasal bridge, and an increased intercanthal distance (Figure 1A). Head appeared enlarged as per age. Head circumference was 51.5 cm (>97 percentile). It was discovered that the remaining anthropometric measurements fell within normal bounds. A biochemical investigation revealed normal bilirubin levels along with increased liver enzymes, including serum alkaline phosphatase 800 IU/L, aspartate transaminase 165 IU/L, and alanine transaminase 42 IU/L. The central region of the brain showed epileptiform discharges on the electroencephalogram.

**Figure 2.**
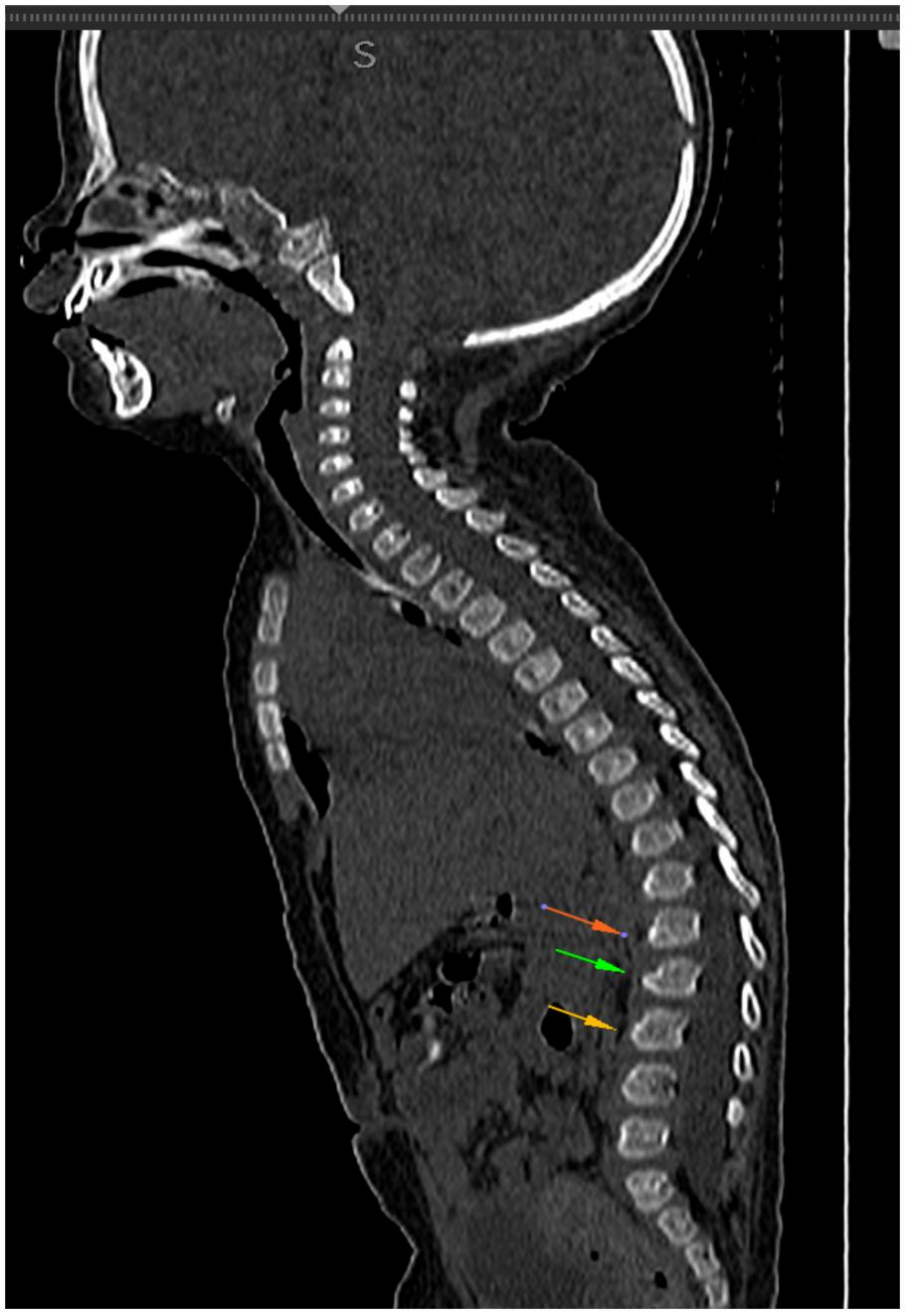
Antero-inferior beaking of second, third, and fourth lumbar vertebrae (arrows).

**Figure 3.**
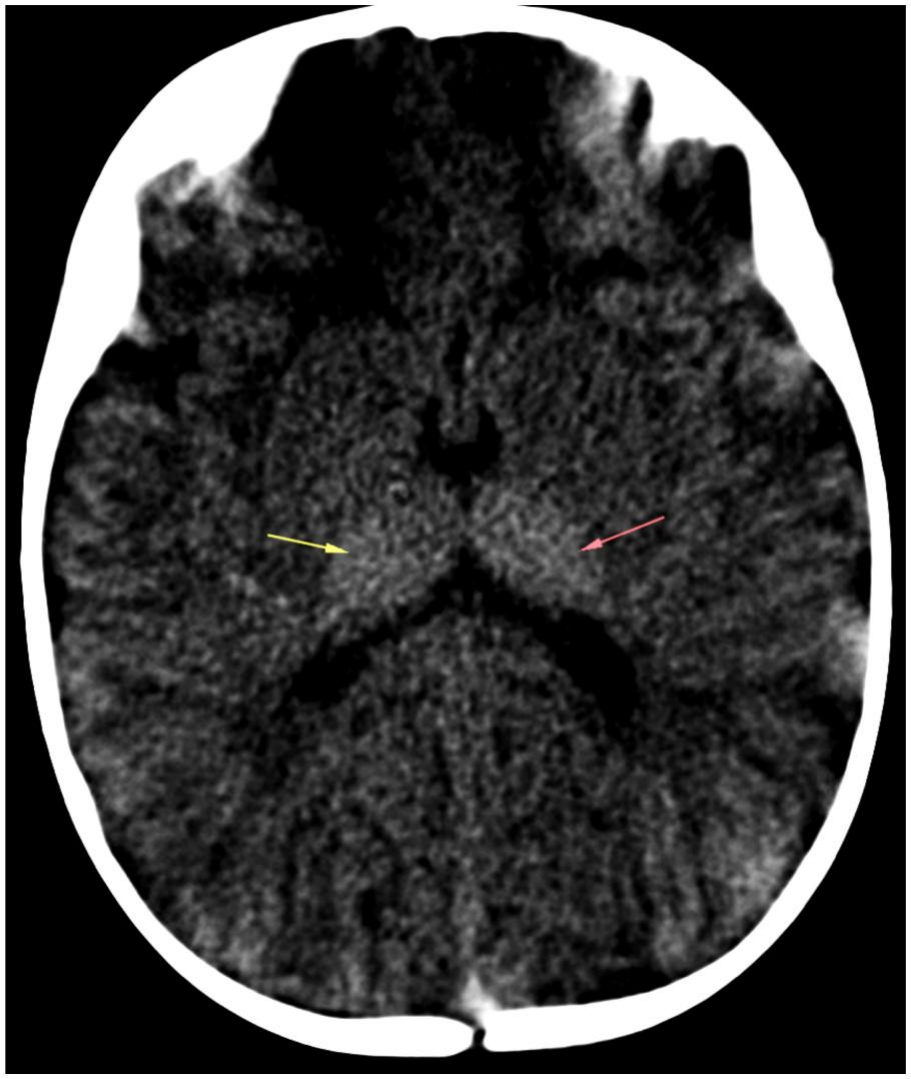
Axial NCCT image demonstrating bilateral hyperdense thalami (arrows). Abbreviation: NCCT = non-contrast CT.

**Figure 4.**
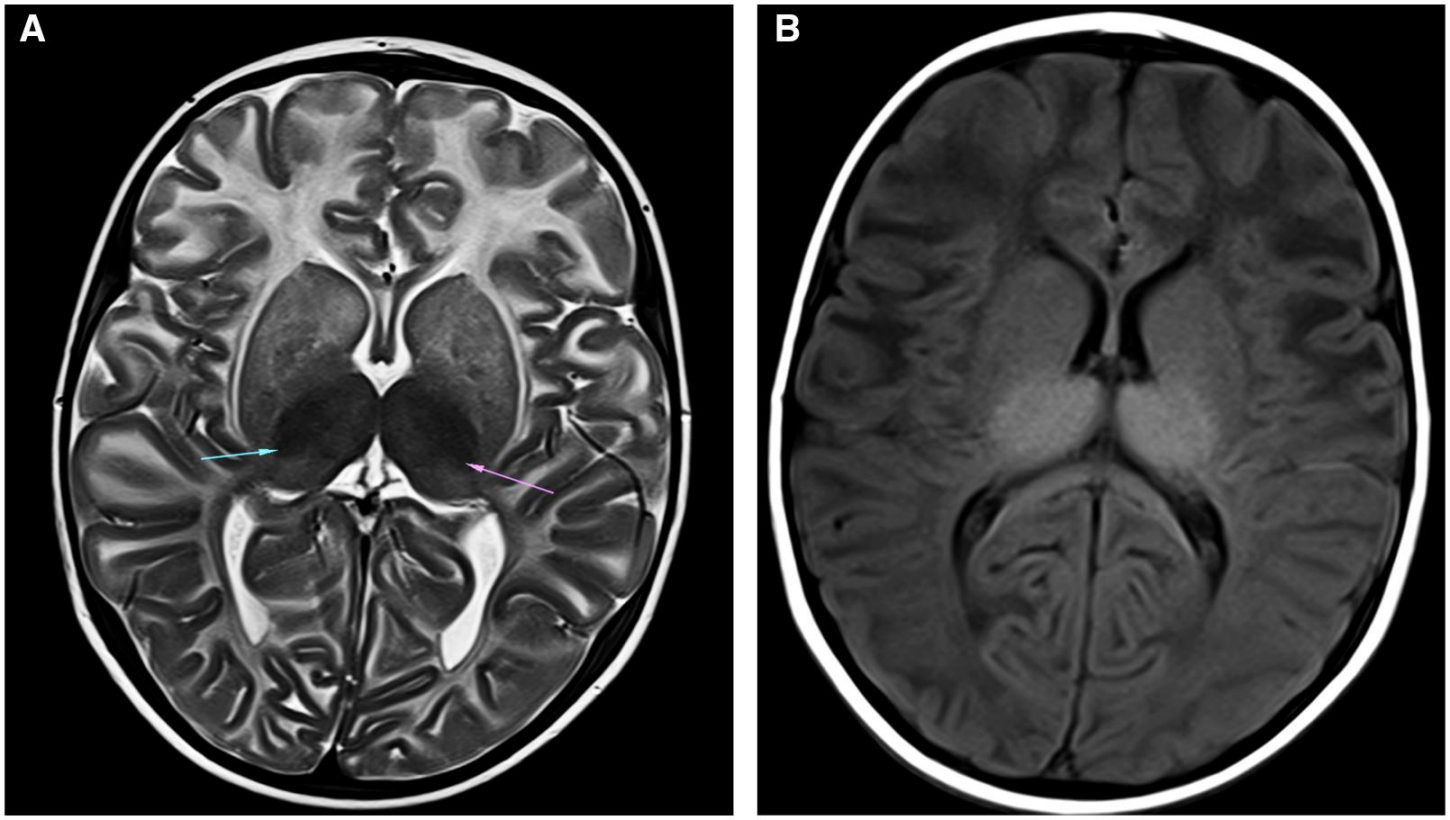
(A) Axial T2-weighted image showing bilateral bulky and hypointense thalami (arrows). (B) Axial T1-weighted image showing bilateral bulky and hyperintense thalami.

**Figure 5.**
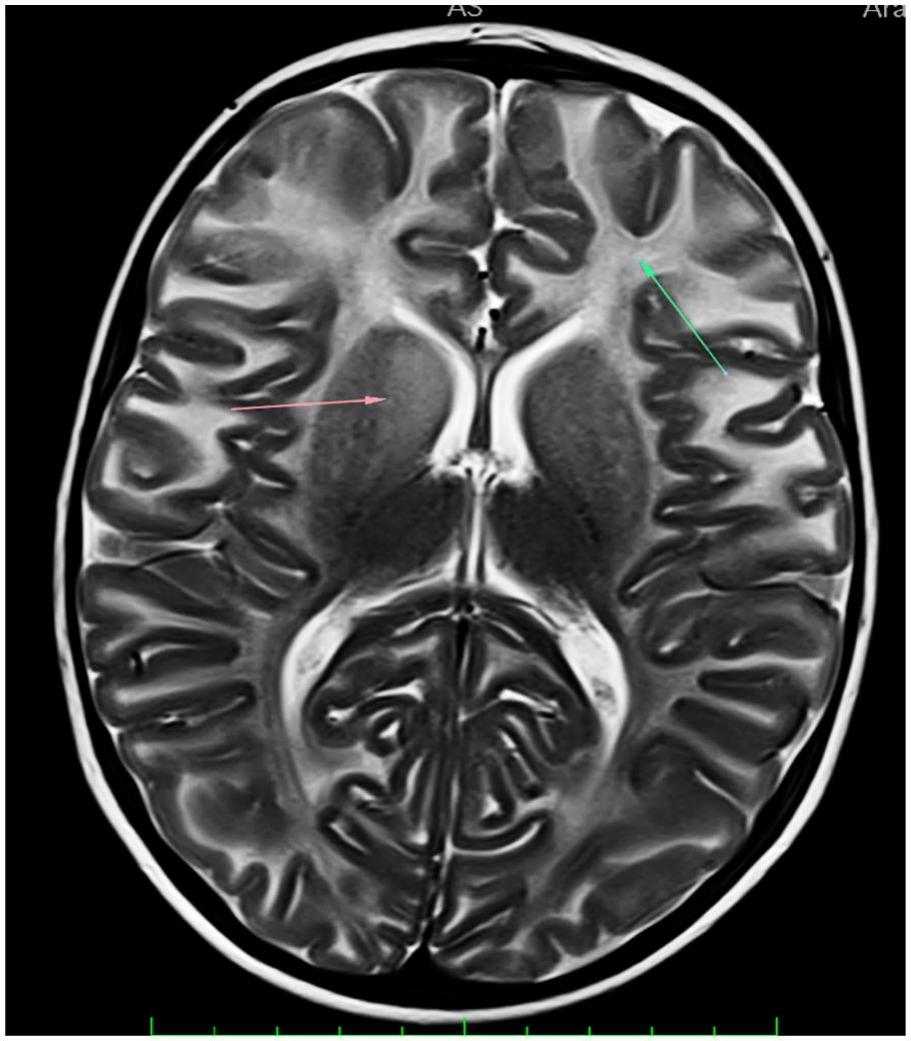
Hyperintensity involving bilateral frontal lobar and periventricular white matter, anterior limb of internal capsule and subcortical U fibres in frontal lobes (arrows).

Sonographic examination demonstrated hepatomegaly, measuring upto 12 cm. Antero-inferior beaking of second, third, and fourth lumbar vertebrae was noted on skeletal survey (Figure 2). Bilateral thalami were hyperdense on CT (Figure 3) and showed T1 hyperintensity and T2 hypointensity (Figure 4A and B). The anterior limb of the internal capsule, bilateral lobar and periventricular white matter, and subcortical U fibres in the frontal lobes show T2 hyperintensity on MRI; the T1 signal alterations were isointense, which suggested hypomyelination (Figure 5). Given the age at presentation, the existence of skeletal and cutaneous abnormalities, and particular MRI abnormalities, these characteristics indicated infantile GM1 gangliosidosis (type 1). The reduction in β-galactosidase activity validated the diagnosis (Tables 1 and 2).

**Table 1.** Genetic index of the infant.

Gene and transcript	Variant	Location	Zygosity	*In silico* parameters	Disorders	Inheritance
GLB1 NM_000404.4	c.385G>C p.Glu129Gln	Exon 3	Heterozygous	CADD: 25.7 SIFT: Deleterious MT: Damaging	GM1-GANGLIOSIDOSIS, TYPE I; GM1G1:230500 GM1- GANGLIOSIDOSIS, TYPE II; GM1G2:230600 GM1- GANGLIOSIDOSIS, TYPE III; GM1G3:230650	Autosomal recessive
GLB1 NM_000404.4	c.289C>T p.Gln97*	Exon 3	Heterozygous	CADD: 36	M1-GANGLIOSIDOSIS, TYPE I; GM1G1:230500 GM1- GANGLIOSIDOSIS, TYPE II; GM1G2:230600 GM1- GANGLIOSIDOSIS, TYPE III; GM1G3:230650	Autosomal recessive

**Table 2.** Summary of the findings.

Summary of findings
The index patient is: Heterozygous for a Likely Pathogenic variant in the *GLB1* gene associated with GM1-GANGLIOSIDOSIS, TYPE I; GM1G1 GM1-GANGLIOSIDOSIS, TYPE II; GM1G2 GM1-GANGLIOSIDOSIS, TYPE III; GM1G3. Heterozygous for a Likely Pathogenic variant in the *GLB1* gene associated with GM1-GANGLIOSIDOSIS, TYPE I; GM1G1 GM1-GANGLIOSIDOSIS, TYPE II; GM1G2 GM1-GANGLIOSIDOSIS, TYPE III; GM1G3

## Discussion

A class of lysosomal storage diseases (LySD) known as gangliosidosis is characterized by lipid buildup in several organ systems. GM1 gangliosidoses, which arises from a lack of the β-galactosidase enzyme, and GM2 gangliosidosis, which arises from a lack of the β-hexosaminidase enzyme, are the 2 primary groups into which they fall.^4^

In GM1gangliosidosis, a rare autosomal recessive lysosomal storage condition, there is neural and visceral accumulation GA1, other minor glycolipids, and glycopeptides due to a lack of the lysosomal enzyme β-galactosidase. Infantile, late infantile/juvenile, and adult are the 3 distinct and age-based clinical phenotypes.^5^

Clinical subtypes of GM1 gangliosidosis are^2^^,^^3^^,^^6^:

1. The infantile form (type 1)—In the initial few days or weeks of infancy, children with type 1 GM gangliosidosis often exhibit hypotonicity and poor head control. Between 3 and 6 months of age, there is a neurological development arrest that frequently leads to feeding issues and underdevelopment. Macular cherry-red patches on ophthalmoscopy, sunken nasal bridge, hypertrophic gums, macroglossia, and “chipmunk face” are examples of dysmorphic characteristics. Within a few months, visual failure occurs.
  Hepatomegaly, splenomegaly, thoraco-lumbar kyphoscoliosis and beaking, subperiosteal new bone development, shorter long bones, and a J-shaped increased sella turcica are among the skeletal radiological findings.
  Thalamic hyperdensity on non-contrast CT (NCCT) scans and hypointense thalami signal with persistently high white matter signal intensity on T2-weighted images are classic neuroimaging findings in patients with infantile GM1gangliosidosis, indicating severely impaired myelination.^5^
  Similar vertebral abnormalities are observed in mucopolysaccharidoses.^7^
  Prognosis is generally poor and death usually occurs by the age of 2 and is frequently brought on by recurrent aspiration pneumonias.
2. Late infantile form/Juvenile (type 2)—Manifests later and is less severe than type I, Between the ages of 12 and 18 months, symptoms begin to appear, such as difficulty in walking or unsteadiness when standing or sitting. Rapid and severe regression causes pseudobulbar symptoms and spastic quadriparesis. The majority of these patients will experience difficult-to-manage epileptic seizures on a regular basis. In contrast to type 1 GM, which exhibits certain dysmorphic traits, there are no dysmorphic features.
  Mild anterosuperior hypoplasia of the vertebral bodies at the thoracolumbar junction is one of the radiographic findings. Globus pallidus MR signal intensity abnormalities and extrapyramidal nucleus paramagnetic ion deposition.
  Prognosis is better as compared to type 1, death usually occurs by second decade.
3. Adult/chronic late-onset form (type 3)—Begins in adulthood, adolescence, or late childhood. These individuals frequently exhibit Parkinsonian symptoms (dysarthria and extrapyramidal signs) along with progressive dementia, which follows typical early cognitive development. It can show variable radiological signs.

Only a small number of patients with type 1 GM1 gangliosidoses have been documented to have neuroimaging findings. In one instance, CT scans revealed an initial thalamic hyperdensity, and later T2-weighted MR images revealed a hypointense signal of the thalami, similar to the findings seen in our case.^8^

Our patient presented with GTCS involving both upper and lower limbs, uprolling of eyes, and drooling of saliva. Additionally, she showed signs of global developmental delay, including chronic bidextrous reach, failure to sit with or without support, and lack of neck holding, all of which were linked to generalized hypotonia. Upon physical examination, the back and gluteal region showed significant cutaneous melanocytosis which has a rare correlation with lysosomal storage disorder.

Only 54 cases of such severe cutaneous melanocytosis linked to lysosomal storage disorders were reported up until 2014, with only 17 of those cases being identified as GM1 gangliosidosis. Similar findings of dermal melanocytosis were noted in our case.

The tyrosine kinase protein is thought to be bounded by the accumulating metabolites in GM1, which raises the amount of nerve growth factors that attach to melanocyte chemotactic receptors, inhibiting melanocyte migration and resulting in widespread cutaneous melanocytosis.^9^

Major differentials for GM1 gangliosidosis include:

1. Krabbe disease
2. Perinatal asphyxia
     (both present with bilateral hyperdense thalami similar to GM1 gangliosidosis)
3. Mucopolysaccharidosis
4. Achondroplasia
5. Congenital hypothyroidism
     (These present as vertebral beaking; however, they show different brain imaging features as compared to GM1 gangliosidosis.)

As of now, the only accessible medical care for the disease is symptomatic and supportive treatment, consisting of maintaining an open airway, eating a healthy diet, staying hydrated, and anti-convulsants to control seizures. Despite attempts at allogenic bone marrow transplantation, the patient’s neurological condition persisted. There is still active research being done on gene therapy and enzyme substitution. Our case demonstrates the common neuroimaging and skeletal findings in this rare disease. Extensive cutaneous melanocytosis contributes to the sparse amount of research on GM1 patients.

## Conclusion

The symptoms of infantile GM1 gangliosidosis (type 1) include a global developmental delay, GTCS, and widespread cutaneous melanocytosis which differentiates it from other cases of diagnosed gangliosidosis and contributes to the sparse amount of research on GM1 patients,

Hepatomegaly, splenomegaly, thoraco-lumbar kyphoscoliosis and beaking, subperiosteal new bone growth, shorter long bones, and a J-shaped increased sella turcica are examples of skeletal radiological characteristics.

Thalamic hyperdensity on NCCT scans and hypointense thalami signal with persistently high white matter signal intensity on T2-weighted images are classic neuroimaging findings in patients with infantile GM1gangliosidosis, indicating severely impaired myelination. These findings will aid with early diagnosis in the future when gene therapy and enzyme replacement therapies become available readily.

## Learning points

- This article gives an insight about infantile GM1 gangliosidosis, a rare genetic lysosomal disorder in infants with its specific clinical features, morphological features, and imaging characteristics.
- Imaging features including antero-inferior beaking of vertebrae, bilateral hyperdense thalami on CT, hypomyelination with T1 hyperintense, and T2 hypointense bilateral bulky thalami were noted on MRI.
- This study would prompt conducting genetic and enzymatic studies and confirmed by decreased β-galactosidase activity.
- Our study demonstrates the typical neuroimaging and skeletal findings in this rare disease.
- Extensive cutaneous melanocytosis seen in our case differentiates it from other cases of diagnoses gangliosidosis and contributes to the sparse amount of research on GM1 patients.
